# Correlation Between Prakriti (Body Constitution) and Severity of Structural Alterations in the Lungs of Patients With SARS-CoV-2: Protocol for a Retrospective Cross-Sectional Study

**DOI:** 10.2196/63916

**Published:** 2026-01-08

**Authors:** Rugaved Gudadhe, Gaurav Sawarkar, Amol Deshpande

**Affiliations:** 1 Department of Rachana Sharir, Mahatma Gandhi Ayurved College Hospital & Research Centre, Datta Meghe Institute of Higher Education, Salod (H), Wardha, Maharashtra, 442001, India, 91 8237434693

**Keywords:** SARS-CoV-2, COVID-19, prakriti, agni, koshtha, structural alterations

## Abstract

**Background:**

SARS-CoV-2, a novel coronavirus, initially appeared in Wuhan, China, at the end of 2019 and has infected more than 31 million people worldwide. Infection can range from asymptomatic to multiorgan failure requiring prompt treatment. Overall, 80% of patients with SARS-CoV-2 experienced mild to moderate illness, while 5% developed serious illness. Physicians can evaluate a patient’s digestive system (*Koshtha*), digestive capacity (*Agni*), strength (*Bala*), and longevity (*Ayu*) using the body constitution (*Deha Prakriti*). It also helps doctors estimate a patient’s illness risk, severity, disease activity scores, and hematological, pathological, and biochemical changes. The study investigated the association between body composition (*Deha Prakriti*) and severity, as shown by structural lung abnormalities in patients with SARS-CoV-2.

**Objective:**

This study aims to study the correlation between the severity of *Prakriti* and structural alterations in the lungs of patients with SARS-CoV-2.

**Methods:**

This is a retrospective cross-sectional study of patients with SARS-CoV-2. The research data will be retrospectively collected from hospital records between September 1, 2020, and May 11, 2021, a period during which India experienced the second wave of the pandemic. The data will come from the Acharya Vinoba Bhave Rural Hospital in Sawangi (Meghe), Wardha, Maharashtra, India. Patients will be contacted via telephone and encouraged to visit the outpatient department and inpatient department at these institutions or in rural and urban areas of Wardha city. A structured case pro forma and a *Prakriti* assessment questionnaire will be used to evaluate lung structural changes during the COVID-19–positive period.

**Results:**

The study is not funded by any organization. The study was initiated on March 4, 2023, and as of July 1, 2024, a total of 265 patients have been recruited. Results will be recorded from the observations of subjective and objective parameters. The study’s primary outcome is to establish a relationship between abnormalities in the structure of the lungs in patients with COVID-19 and body constitution (*Prakriti*). The study’s secondary outcome will help identify which body constitution is most susceptible to structural changes and disease severity in patients with COVID-19 and will also offer insights into preventive medicine.

**Conclusions:**

Statistical investigation will lead to the conclusion that there is a specific association between structural abnormalities in the lungs of patients with COVID-19 and their body constitution. We hypothesize that *Prakriti* will be identified as being more prone to structural changes and severity in patients with COVID-19, offering insights into preventive therapy.

## Introduction

### Background

The appearance and spread of the 2019 novel COVID-19 or SARS-CoV-2 has caused a new public health disaster that is putting the entire world at risk [1]. As the SARS-CoV-2 virus causes COVID-19, it is commonly referred to as a viral respiratory and vascular disorder [2]. COVID-19 causes pneumonia similar to Middle East respiratory syndrome and SARS [3,4]. The clinical signs range from fever, headaches, and myalgia symptoms, which are common with viral pneumonia, which can result in severe respiratory failure and death [5,6]. Ground glass opacities and consolidation, primarily in the lower lobe and peripheral regions, are common computed tomography (CT) findings of COVID-19 pneumonia. Other findings include a crazy paving pattern, vascular enlargement, an air bubble sign, halo and reverse halo signs, airway changes, and airway alterations in bronchograms [7,8]. Atypical observations include the following traits: central distribution, peri-broncho-vascular dissemination, isolated involvement of the upper lobe, lobar and segmental consolidation, nodules, subpleural sparing, pleural and pericardial effusion, and white lung [9-11]. According to the findings, the most common lesion patterns on thorax CT scans were ground-glass opacity and consolidation. There were also unusual pavement patterns, airway changes, reverse halo signs, halo signs, and air bubble signs [12,13].

Patients’ CT thorax examinations frequently reveal the following lesion patterns: (1) ground-glass opacity and (2) consolidation. Following COVID-19’s acute phase, pulmonary fibrosis develops as the second serious respiratory consequence. Patients with post-acute COVID-19 syndrome frequently have dyspnea, cough, oxygen dependence, difficulty weaning from mechanical ventilation or noninvasive ventilation, changes to their fibrotic lungs, decreased diffusion capacity, and decreased endurance [14]. Regardless of viral status, the term “Long COVID” refers to the presence of a variety of symptoms weeks or months after SARS-CoV-2 infection. It is also known as “post-COVID syndrome.” Its pattern can be chronic or relapsing-remitting [15,16]. People with “long COVID” frequently experience symptoms, such as severe exhaustion, breathlessness, coughing, chest pain, palpitations, headache, joint pain, myalgia, weakness, insomnia, diarrhea, rash, hair loss, impaired balance and gait, neurocognitive issues, such as memory and concentration issues, and a lower quality of life. “Long COVID” patients may have 1 or more symptoms [17]. Chronic cough, fibrotic lung disease (post–COVID-19 fibrosis or post-acute respiratory distress syndrome fibrosis), bronchiectasis, and pulmonary vascular disease are some of the pulmonary issues that can develop as a result of SARS-CoV-2 infection [15]. Chronic shortness of breath may result from persistent pulmonary involvement, which usually resolves over time. Unfortunately, CT scans of many COVID-19 asymptomatic patients show severe lung involvement. COVID-19–induced lung fibrosis may cause persistent dyspnea and necessitate additional oxygen [18]. People infected with SARS-CoV-2 face significant social and economic consequences if their chronic symptoms persist. As the illness spreads, more people may require medical attention, putting pressure on the health care system [19]. It also has an impact on one’s quality of life; therefore, the concept of personalized medication may be more advantageous. Ayurvedic treatment will be based on body constitution (*Prakriti*) in a similar vein, as it outlines the treatment plan and will aid in effective management. Body constitution (*Prakriti*) (air entity [*Vata*]*,* hot entity [*Pitta*], and phlegm [*Kapha*]*,* the 3 functional constituents of the body) is the formation of distinctive qualities caused by the dominance of *Doshas* in this sense.

### Rationale for the Study

Body constitution (*Prakriti*) refers to an individual’s predominance of 1 or more *Doshas*. Other factors also have an impact on a body’s composition [20]. The ancient books of Ayurveda also include recommendations for maintaining a lifestyle per one’s body constitution (*Prakriti*) to continue healthy living in a personalized manner [21]. Body constitution (*Prakriti*) refers to specific physical and mental characteristics that are associated with disease susceptibility. We also anticipate that it will be possible to genotypically screen newborns to identify their body constitution (*Prakriti*), which will help us choose the type of healthy lifestyle that will allow these newborns to live long, disease-free lives in the future. This will be a groundbreaking development for personalized preventative medicine in humans [22]. Ayurveda has provided explicit instructions in the form of a daily routine (*Dinacharya*) and a seasonal daily routine (*Rutucharya*) to maintain the associated with *Dosha,* the natural functioning of a specific body constitution (*Prakriti*) [23]. According to Ayurveda, body constitution (*Prakriti*) plays an important role in disease susceptibility as well as the selection of preventative and therapeutic methods. Notably, a person’s body constitution (*Prakriti*) influences how their immune system (*Bala*) functions [24,25]. The study’s goal is to investigate the relationship between body constitution (*Prakriti*) and severity as evidenced by structural changes in the lungs of patients with SARS-CoV-2.

### Objectives

This research aims to investigate structural changes in the lungs of patients with SARS-CoV-2, assess their body constitution (*Prakriti*), and correlate structural changes in the lungs of patients with SARS-CoV-2 with their body constitution (*Prakriti*).

## Methods

### Overview

This is a retrospective cross-sectional study of patients with SARS-CoV-2. The research data will be retrospectively collected from hospital records between September 1, 2020, and May 11, 2021, as India experiences the second wave of the pandemic during this period. The data will come from the Acharya Vinoba Bhave Rural Hospital in Sawangi (Meghe), Wardha, Maharashtra, India. Patients will be contacted via telephone and encouraged to visit the outpatient department and inpatient department at these institutions or in rural and urban areas of Wardha city. A structured case pro forma and a *Prakriti* assessment questionnaire will be used to evaluate lung structural changes during the COVID-19–positive period (Multimedia Appendix 1).

### Sample Calculation

Sample calculation will be done according to the following formula:


$$\frac{n\geq Z_{1-\frac{a}{2}}^{2}\times P\times (1-p)}{d^{2}}$$

### Sample Size

The sample size is calculated as 246 based on the proportion or prevalence of ground glass opacity at 80% (0.8) and Z alpha at 5% level (1.96 constant), with an estimated margin of error of 5% (0.05) [26]. The formula for calculating the sample size was N=(1.96)2 × 0.8 × (1–0.2)/(0.05)2=246 minimum

### Grouping

The study will include 2 groups, each consisting of 246 individuals. Group A comprises individuals exposed to SARS-CoV-2 with lung involvement, while Group B includes those exposed to the virus without lung involvement. This classification aims to assess the impact of SARS-CoV-2 on lung health and compare outcomes between affected and unaffected individuals. Understanding these differences can help in evaluating disease severity, treatment approaches, and long-term health implications for patients with and without pulmonary complications due to the virus. Group A will include 246 participants exposed to SARS-CoV-2 having lung involvement, and Group B will include 246 participants exposed to SARS-CoV-2 having no involvement of the lung.

### Assessment Parameter

#### Reverse Transcription–Polymerase Chain Reaction

Reverse transcription–polymerase chain reaction (RT-PCR) is the standard test for detecting SARS-CoV-2. It is typically performed on a sample of respiratory or nasopharyngeal secretions. Although RT-PCR is thought to be quite specific, its sensitivity might vary from 60%‐70% to 95%‐97% [27].

#### Chest X-Ray

It is helpful in the assessment of the structural changes in the lung field and identification of potential side effects, including pneumothorax, subcutaneous emphysema, and pneumo-mediastinum, and ongoing tracking of the progression of the illness. The most frequent discoveries are opacities in the airspace, which might be either consolidations or, less frequently, opacities in the ground glass [27].

#### High-Resolution Computed Tomography—Chest

It is a quick, accessible test considered to be the most sensitive imaging test for detecting COVID-19, with a reported sensitivity of up to 97%. Typical findings include ground glass opacities, consolidation, peripheral reticulation, and a crazy paving pattern [27].

The patient’s body constitution (*Prakriti*) will be assessed by the personal interviewing method. The standard pro forma for the assessment of *Prakriti* is attempted to develop according to the Central Council for Research in Ayurvedic Sciences’ *Prakriti* assessment module to ensure physical constitution of the human body based on several views of the body constitution [28].

### Inclusion Criteria

Nonvaccinated patients with SARS-CoV-2, regardless of age or gender, who tested positive for RT-PCR between September 1, 2020, and May 11, 2021, and were willing to provide informed consent will be included in the study.

### Exclusion Criteria

The study will exclude patients with respiratory disorders, such as chronic obstructive pulmonary disease and bronchial asthma, and those with multiple organ disorders. For more than a year, patients who smoked more than 2 cigarettes per day and consumed more than 60 mL of alcohol per day were also excluded.

### Data Sources and Management

#### Overview

Patient data will be screened from the medical record department and patients with post–COVID-19 (who tested positive within the specified time period) at Acharya Vinoba Bhave Rural Hospital Sawangi’s outpatient department and inpatient department. Personal interviews and assessments will be conducted to determine the body’s constitution (*Prakriti*).

#### Gradation and Calculation of *Prakriti*

The final percentage scores for *Vata*, *Pitta*, and *Kapha* are determined using the formula:


$$Final\;percentage\;scores\;for\;Vata,\;Pitta,\;and\;Kapha=(\frac{The\;overall\;points\;obtained\;by\;an\;individual\;for\;a\;Dosha}{The\;total\;points\;assigned\;to\;Dosha})\times 100$$

The collected data will be categorized and fractionalized using frequencies and percentages [29,30].

### Statistical Analysis

The body constitution (*Prakriti*) will be evaluated, and the resulting data will be classified and fractionalized using frequency and percentages. Following that, the chi-square test will be used in the study.

### Ethical Considerations

This research obtained approval from the Institutional Ethics Committee of Datta Meghe Institute of Medical Sciences (deemed to be a University) on August 1, 2022, with reference letter MGACHRC/IEC/Aug-2022/558. The purpose of the study will be explained to the participants and their guardians in the local language, with a pro forma designed for this purpose. After bringing all these to the informant’s notice, data will be collected with written informed consent, keeping the information confidential by interviewing them afterwards. Participants’ information will be deidentified, with personal identifiers stored separately from research data. Data will be retained for a specific duration and securely disposed of thereafter. Study procedures will be conducted privately, and no identifiable information will be disclosed without explicit consent. The study will comply with applicable data protection regulations, and participants will be informed of confidentiality measures through the consent process. Participants will not incur any financial costs for participating in this study. If applicable, compensation will be provided. In the unlikely event of any adverse effects or injury directly caused by the study, appropriate medical care and compensation will be provided in accordance with institutional and regulatory guidelines. Participants will be informed about their rights and the compensation process during the consent process.

## Results

**Figure 1.**
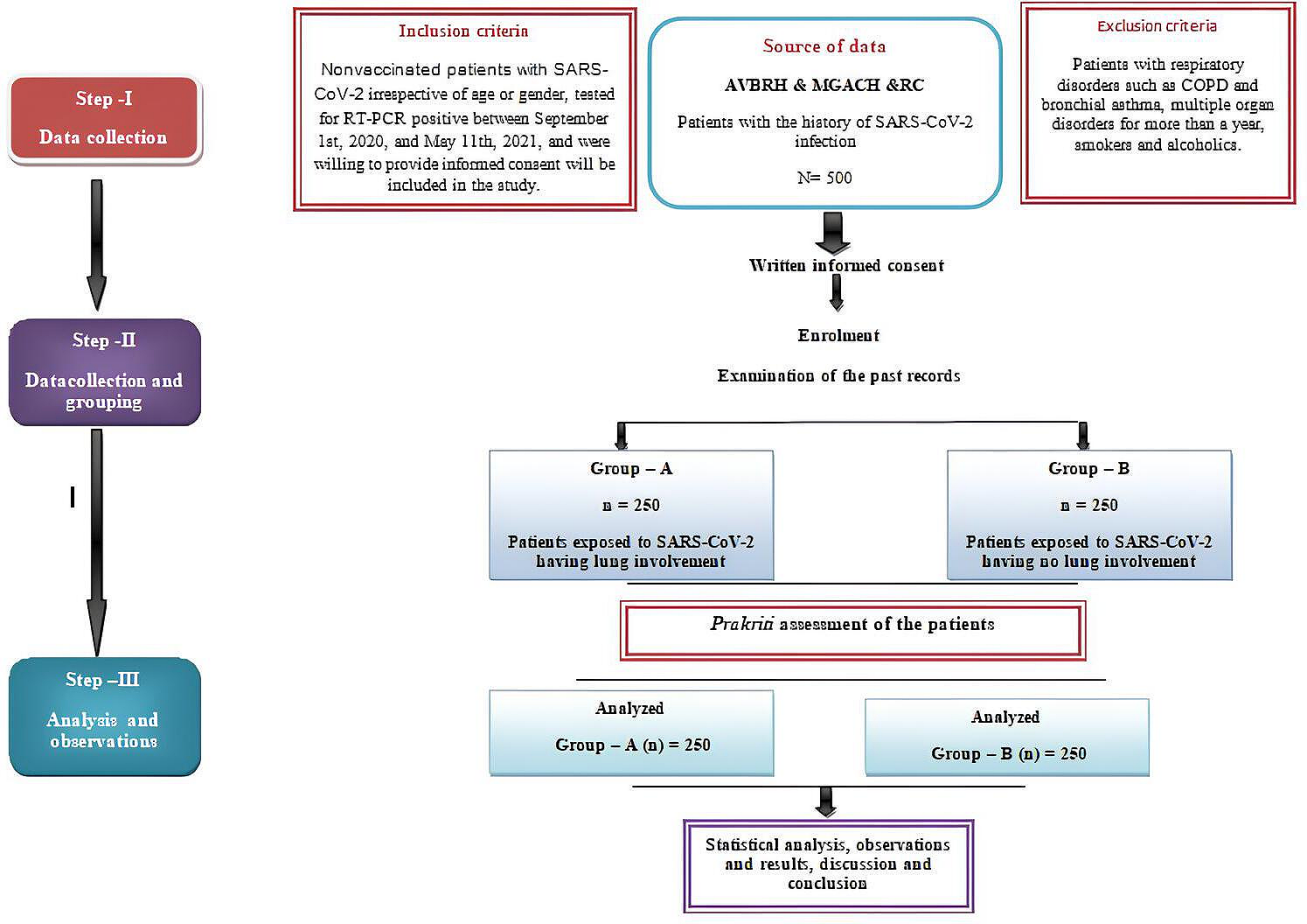
Study design. AVBRH: Acharya Vinoba Bhave Rural Hospital; COPD: chronic obstructive pulmonary disease; MGACH & RC: Mahatma Gandhi Ayurved College Hospital and Research Centre; RT-PCR: reverse transcription–polymerase chain reaction.

The study was initiated in March 2023, and as of July 2024, a total of 265 patients have been recruited. Results will be recorded from the observations of subjective and objective parameters. The study’s primary outcome is to establish a relationship between abnormalities in the structure of the lungs of patients with COVID-19 and body constitution. The study’s secondary outcome will provide a better understanding of which body constitution is most susceptible to structural changes and disease severity in patients with COVID-19 and will also offer insights relevant to preventive medicine (Figure 1).

## Discussion

### Expected Findings

This study investigates the correlation between structural lung changes and different types of *Prakriti* in patients with SARS-CoV-2. If a significant relationship is found, it could support Ayurveda-based predictive models for disease severity. While prior research has focused on clinical and radiological factors, this study uniquely integrates traditional medicine with modern diagnostics. Strengths include its novel approach, though variability in *Prakriti* assessment may pose challenges. Future research should expand on these findings to enhance personalized health care strategies.

### Importance of Body Constitution (*Deha Prakriti*) in Disease Susceptibility

A person’s susceptibility to a specific illness can be determined by examining their body constitution (*Prakriti*), which is the key. According to Ayurvedic literature, the *Deha Prakriti* can affect the disease’s susceptibility and severity. According to the literature, a person is more susceptible to a disease caused by the same *Dosha* in his body constitution (*Prakriti*) if the etiology is strong and comparable. Compared to people with other body constitutions (*Deha Prakriti*), those with a prominent *Kapha Dosha* are more quickly and easily impacted by powerful etiological variables that aggravate their *Kapha Dosha*. Similarly, if a person with *Vataja Prakriti* follows a diet and daily habits (*Ahara-Vihara*) that promote *Vata*, they are more likely to develop *Vataja* illness, which is difficult to treat due to the dominant *Dosha* of their body constitution (*Prakriti*). They experience this if they follow a *Vata*-inducing diet and daily habits (*Ahara-Vihara*). This belief holds that people with *Vataja Prakriti* are more susceptible to ailments caused by vitiated *Vata. Pittaja Prakriti* people are more likely to experience *pittaja* specialized difficulties, as are *Kaphaja* people. *Vata*, *Pitta*, and *Kapha* constitutions (*Prakritis*) have a lower risk of developing various ailments as their body immunity (*Bala*) and life span (*Ayu*) develop [31].

### Importance of *Deha Prakriti* in Disease Prognosis

Using *Deha Prakriti* analysis, you can predict how a disease will develop. Unlike *Deha Prakriti,* the illness known as curable disease (*Sadhya Vyadhi*), which affects etiology (*Hetu*), premonitory symptoms (*Purvaroopa*), and signs and symptoms (*Rupa*), causes weakness (*Balakshaya*). When one of the body’s constitutions (*Prakriti*), the vitiated part (*Dushya*), or period (*Kala*) resembles the *Vikarjanak Dosha*, and *Hetu*, *Purvaroopa*, and *Rupa* are moderate members, healing is difficult (*Kashtasadhya*) [31]. Only a small percentage of patients who recover from COVID-19 experience the illness known as “long COVID,” “Long Haulers,” or “Post COVID syndrome,” which is characterized by additional or persistent symptoms that last for weeks or months [15]. SARS-CoV-2 infection may cause bronchiectasis, post–COVID-19 fibrosis, chronic cough, and pulmonary vascular disease [17]. Unfortunately, many CT scans of asymptomatic patients with COVID-19 show severe lung disease. COVID-19 in lung fibrosis increases the risk of chronic dyspnea and necessitates the use of additional oxygen [32]. SARS-CoV-2 infection symptoms that last for an extended period of time have significant social and economic consequences. As the illness spreads, more people may require medical care in the near future, putting an unfair strain on the health care system. Providing medical professionals with specific instructions for managing chronic COVID-19 will help reduce confusion. More data will be obtained by tracking patients who have recovered from COVID-19 over time [33].

### Conclusions

Statistical investigation will lead to the conclusion that there is a specific association between structural abnormalities in the lungs of patients with COVID-19 and their body constitution. We hypothesize that *Prakriti* will be identified as being more prone to structural changes and severity in patients with COVID-19, offering insights into preventive therapy.

## Supplementary material

10.2196/63916 Multimedia Appendix 1 Case paper for *Prakriti* assessment.

## Abbreviations

CT: computed tomography
IPD: inpatient department
OPD: outpatient department
RT-PCR: reverse transcription–polymerase chain reaction
