# Supplementary material for: Correlation Between Prakriti (Body Constitution) and Severity of Structural Alterations in the Lungs of Patients With SARS-CoV-2: Protocol for a Retrospective Cross-Sectional Study
Source: JMIR Res Protoc. 2026 Jan 8;15:e63916. doi: 10.2196/63916 (PMC12782462; doi:10.2196/63916)
Supplement: Multimedia Appendix 1 [file resprot-v15-e63916-s001.pdf]

Case sheet

CASE PAPER

IPD NO. - __________

AGE- __________

SEX- MALE/FEMALE/ OTHER

FINAL DIAGNOSIS-_____________________

DATE OF ADMISSION-_____________________

INCLUSION TEST - 1. RTPCR

2. COVID ANTIGEN TEST

HRCT SCORE- ______________

STRUCTURAL CHANGES IN HRCT-_______________________________

X-RAY CHEST- _______________________________

SMOKER / ALCOHOLIC- YES/NO

BRONCHIAL ASTHAMA / COPD- YES/NO

PRAKRITI ASSESSMENT FORM

Q-1. Body structure

- ***Vataj***: Short, thin, weak
- ***Pittaj***: Medium, fleshy, delicate
- ***Kaphaj***: In proportion, well built, complete, beautiful

Q-2. Body frame

- ***Vataj***: Lean ,short
- ***Pittaj***: Medium, plump
- ***Kaphaj***: Large, well built

Q-3. Hair: body hair, beard or moustache

- ***Vataj***: Scanty, dry, splitting
- ***Pittaj***: Scanty, soft, tendency to grey hair & baldness
- ***Kaphaj***: Plentiful, wavy, glossy, do not fall or grey early

Q-4. Hair: Colour

- ***Vataj***: Dusky hair
- ***Pittaj***: Reddish brown hair
- ***Kaphaj***: Black hair

Q-5. Skin

- ***Vataj***: Dry, cracking,rough, black coloured
- ***Pittaj***: Oily, soft with moles, pimples, freckles
- ***Kaphaj***: Glossy, clear, smooth, glorious, fair, pinkish

Q-6. Nails

- ***Vataj***: Small, blackish, cracking, breaking
- ***Pittaj***: Small, reddish, smooth, flat
- ***Kaphaj***: Big, pinkish, smooth, glossy, convex

Q-7. Lips

- ***Vataj***: Blackish, cracking, shapeless
- ***Pittaj***: Reddish, smooth, thin, soft
- ***Kaphaj***: Pinkish, smooth, glossy, proportionate

8. Teeth

- ***Vataj***: Very small or very big, cracking, brocken, irregular
- ***Pittaj***: Medium size with gap
- ***Kaphaj***: Even, glazing , straight, smooth, shiny, white

9. Eyes shape and Colour

- ***Vataj***: Small, round eyes blackish sclera, unsteady eyes, habit of raising eyebrows
- ***Pittaj***: White part reddish, brown eyes , reddish canthus of eyes sharp eyesight
- ***Kaphaj***: Big eyes Elongated milky white sclera, edges reddish, calm, soft, gentle look

10. Eye Lashes

- ***Vataj***: Thin and less eye lashes
- ***Pittaj***: Less in number/ Thin eye lashes
- ***Kaphaj***: Thick/Dense eye lashes

11. Eyes movements

- ***Vataj***: Eyes remains half closed during sleep
- ***Pittaj***: Eyes get redden during anger /Alcohol consumption/Exposure to light
- ***Kaphaj***: Steady gaze/Less blinking

12. Body temperature

- ***Vataj***: Less than that of normal, palms and feet are cold
- ***Pittaj***: More than that of normal; palms, feet, face and forehead are hot
- ***Kaphaj***: Normal; palms and feet are slightly cold

13. Joints

- ***Vataj***: Protuberant, cracking, unsteady
- ***Pittaj***: Loose, moderately hidden
- ***Kaphaj***: Strong, firm, compact, well hidden

14. Muscles

- ***Vataj***: Prominent/ Hard calf muscle
- ***Pittaj***: Lax muscles
- ***Kaphaj***: Well built muscles

15. Gait

- ***Vataj***: Speedy/Swift gait/ Small steps, Quick initiation
- ***Pittaj***: Moderate/Normal steps
- ***Kaphaj***: Steady gait/Firm steps

16. Voice

- ***Vataj***: Rough, hoarse, weak, dry
- ***Pittaj***: Clear, loud
- ***Kaphaj***: Like lion, mridanga, deep, pleasant, soft

17. Body movements

- ***Vataj***: Unsteady, like to wander here and there, habit of moving hands, legs and shoulders
- ***Pittaj***: Fast movements
- ***Kaphaj***: Slow, steady

18. Sweat

- ***Vataj***: Nothing particular/ less sweating
- ***Pittaj***: Very easily and a lot, in armpits, head, the body emits the foul smell and sweat
- ***Kaphaj***: Less sweating

19. Appetite

- ***Vataj***: Unpredictable, sometimes good, sometimes less, like to it fast
- ***Pittaj***: Good, has to eat after three to four hours, cannot tolerate hunger/thirst
- ***Kaphaj***: Comparatively less, can tolerate hunger/thirst easily

20. Thirst

- ***Vataj***: Frequently thirsty/ Drinks more water
- ***Pittaj***: Drinks more water/Intense thirst
- ***Kaphaj***: Drinks less water/Less thirst

21. Quantity of food and drinks consumed

- ***Vataj***: Not fixed, sometime more sometime less
- ***Pittaj***: Comparatively more
- ***Kaphaj***: Comparatively less

22. Food preferred

- ***Vataj***: Hot and wet
- ***Pittaj***: Cold and liquid
- ***Kaphaj***: Hot and dry

23. Eating habit

- ***Vataj***: Fast eating habit
- ***Pittaj***: Moderate
- ***Kaphaj***: Slow eating habit

24. Stools

- ***Vataj***: Blackish, hard, once in a day
- ***Pittaj***: Yellowish, loose, once/twice a day
- ***Kaphaj***: Yellowish, well formed, once in a day

25. Sleep

- ***Vataj***: Less than six hours, interrupted
- ***Pittaj***: Six to eight hours, sound sleep
- ***Kaphaj***: Eight hours or more, sound sleep

26. Dream

- ***Vataj***: Skies, winds, hurricae, hollowness etc
- ***Pittaj***: Fire, lightning, gold, red/yellow
- ***Kaphaj***: Water, pools, gardens, faint coloured views

Total-

- ***Vata-***
- ***Pitta***-
- ***Kapha***-

Percentage-

- ***Vata -*** _______
- ***Pitta-*** _______
- ***Kapha-*** _______

Prakriti- __________
